# CNN-Based LCD Transcription of Blood Pressure From a Mobile Phone Camera

**DOI:** 10.3389/frai.2021.543176

**Published:** 2021-05-21

**Authors:** Samruddhi S. Kulkarni, Nasim Katebi, Camilo E. Valderrama, Peter Rohloff, Gari D. Clifford

**Affiliations:** ^1^School of Electrical and Computer Engineering, Georgia Institute of Technology, Atlanta, GA, United States; ^2^Department of Biomedical Informatics, Emory University, Atlanta, GA, United States; ^3^Wuqu' Kawoq | Maya Health Alliance, Chimaltenango, Guatemala; ^4^Division of Global Health Equity, Brigham and Women's Hospital, Boston, MA, United States; ^5^Department of Biomedical Engineering, Georgia Institute of Technology & Emory University, Atlanta, GA, United States

**Keywords:** blood pressure, convolutional neural network, digital transcription, hypertension, optical character recognition, preeclampsia

## Abstract

Routine blood pressure (BP) measurement in pregnancy is commonly performed using automated oscillometric devices. Since no wireless oscillometric BP device has been validated in preeclamptic populations, a simple approach for capturing readings from such devices is needed, especially in low-resource settings where transmission of BP data from the field to central locations is an important mechanism for triage. To this end, a total of 8192 BP readings were captured from the Liquid Crystal Display (LCD) screen of a standard Omron M7 self-inflating BP cuff using a cellphone camera. A cohort of 49 lay midwives captured these data from 1697 pregnant women carrying singletons between 6 weeks and 40 weeks gestational age in rural Guatemala during routine screening. Images exhibited a wide variability in their appearance due to variations in orientation and parallax; environmental factors such as lighting, shadows; and image acquisition factors such as motion blur and problems with focus. Images were independently labeled for readability and quality by three annotators (BP range: 34–203 mm Hg) and disagreements were resolved. Methods to preprocess and automatically segment the LCD images into diastolic BP, systolic BP and heart rate using a contour-based technique were developed. A deep convolutional neural network was then trained to convert the LCD images into numerical values using a multi-digit recognition approach. On readable low- and high-quality images, this proposed approach achieved a 91% classification accuracy and mean absolute error of 3.19 mm Hg for systolic BP and 91% accuracy and mean absolute error of 0.94 mm Hg for diastolic BP. These error values are within the FDA guidelines for BP monitoring when poor quality images are excluded. The performance of the proposed approach was shown to be greatly superior to state-of-the-art open-source tools (Tesseract and the Google Vision API). The algorithm was developed such that it could be deployed on a phone and work without connectivity to a network.

## 1. Introduction

Over half a million women die each year from pregnancy-related causes, and the vast majority of these deaths occur in low- and middle-income countries (LMICs) (WHO, 2005). Despite global improvements in healthcare, countries with lowest GDP per capital have made little progress and shoulder the vast majority of the global burden for fetal and maternal mortality and morbidity. There is, therefore, a critical need to focus on low-cost screening and community-based interventions to reduce preventable maternal and fetal mortality and morbidity (Salam et al., 2015).

Hypertensive disorders during pregnancy are a significant contributor to this perinatal morbidity and mortality (Khan et al., 2006). It has been reported that 10% of women have high blood pressure during pregnancy, and preeclampsia complicates 2–8% of all pregnancies (Duley, 2009). In LMICs, results showed that overall 10–15% of direct maternal deaths are associated with preeclampsia (Khan et al., 2006; Duley, 2009). Hypertensive disorders of pregnancy includes chronic hypertension, gestational hypertension, and preeclampsia, and there is substantial evidence that these lead to adverse outcomes both during pregnancy and after birth (Berends et al., 2008; Rich-Edwards et al., 2010; Melchiorre et al., 2011; Mosca et al., 2011; Powers et al., 2012; Catov et al., 2013; Parikh et al., 2017; Whelton et al., 2018; Haas et al., 2019; Bergman et al., 2020; Boardman et al., 2020; Gooding et al., 2020; Scheres et al., 2020; Wu et al., 2020).

In particular, diverse outcomes related to hypertensive disorders of pregnancy can affect both mother and fetus in long and short term. They are associated with placental abruption, preterm delivery, fetal growth restriction, stillbirth, maternal death secondary to stroke and preeclampsia, as well as future risk of hypertension, diabetes mellitus, and cardiovascular disease in the mother (ACOG, 2013). Moreover, blood pressure monitoring and management, has been shown to be beneficial during pregnancy (Scantlebury et al., 2013; Gillon et al., 2014; Magee et al., 2016; Podymow and August, 2017; Chawla et al., 2020; Whybrow et al., 2020). However, the majority of evidence is provided for populations in high-income settings. As Salam et al. (2015) noted, there is a need to improve low-cost screening of blood pressure and interventions for hypertensive disorders of pregnancy in LMICs, and to control preeclampsia in particular. This is expected to have a significant impact in preventing maternal and fetal mortality. The authors suggest the need to invest more in research at primary care level to improve the evidence base for community-level interventions.

Although numerous clinical and biochemical tests have been proposed for prediction or early detection of preeclampsia, most remain unrealistic for general use in LMICs (Wagner, 2004; Osungbade and Ige, 2011). Challenges in the management of preeclampsia in low-resource settings include failure to identify preeclampsia along with a delay in responding to the clinical signs and symptoms due to the limited access to health care centers. For these reasons, routine blood pressure measurement in pregnancy is essential in the antenatal period. Therefore, designing low-cost and accessible monitoring systems, along with decision support, is essential to improving the quality of pregnancy care in LMICs and improving patient outcomes.

While blood pressure monitoring is a key component to monitoring maternal-fetal well-being during pregnancy, it is important to note that it is also prone to errors through incorrect usage, poor choice of device and arm cuff, poor body habitus, and transcription or transmission errors (Mishra et al., 2013). In a related work, the authors demonstrated that even trained clinical experts make significant errors when transcribing basic medical information (Hall-Clifford et al., 2017). In the same work, they also showed that capture of data by mobile phones and tablets enabled them to reduce errors significantly, with only poor handwriting (that even the authors could not recognize at times, or remember writing), remaining as the barrier to accurate record keeping.

A number of wireless BP devices are now commercially available, with data transmission almost exclusively based on some variant of Bluetooth. Wireless handshaking is prone to connectivity errors due to radiofrequency interference, variations in standards, and non-causal activity on the phone (with various installed apps and services interfering with the connection). More importantly, to the best of the authors' knowledge, no BP devices with wireless connectivity have been validated in a preeclamptic population. The definitive work evaluating devices in such a population was performed by Bello et al. (2018), who identified only a very small number of devices which are appropriate for preeclampsia, and none with wireless connectivity. This presents a key problem for monitoring BP in pregnancy. Moreover, given the volume of legacy medical devices around the world which lack wireless connectivity, it is important that there is an efficient and reliable method for transcribing, reading, and transmitting data from standard BP devices. The virtually ubiquitous cellphone camera provides a potential scalable solution through optical character recognition (OCR). To date, there is no study evaluating the effectiveness of BP digitization, and its conformity with acceptable standards for use in clinical diagnosis, particularly for use in pregnancy.

**Figure 1:**
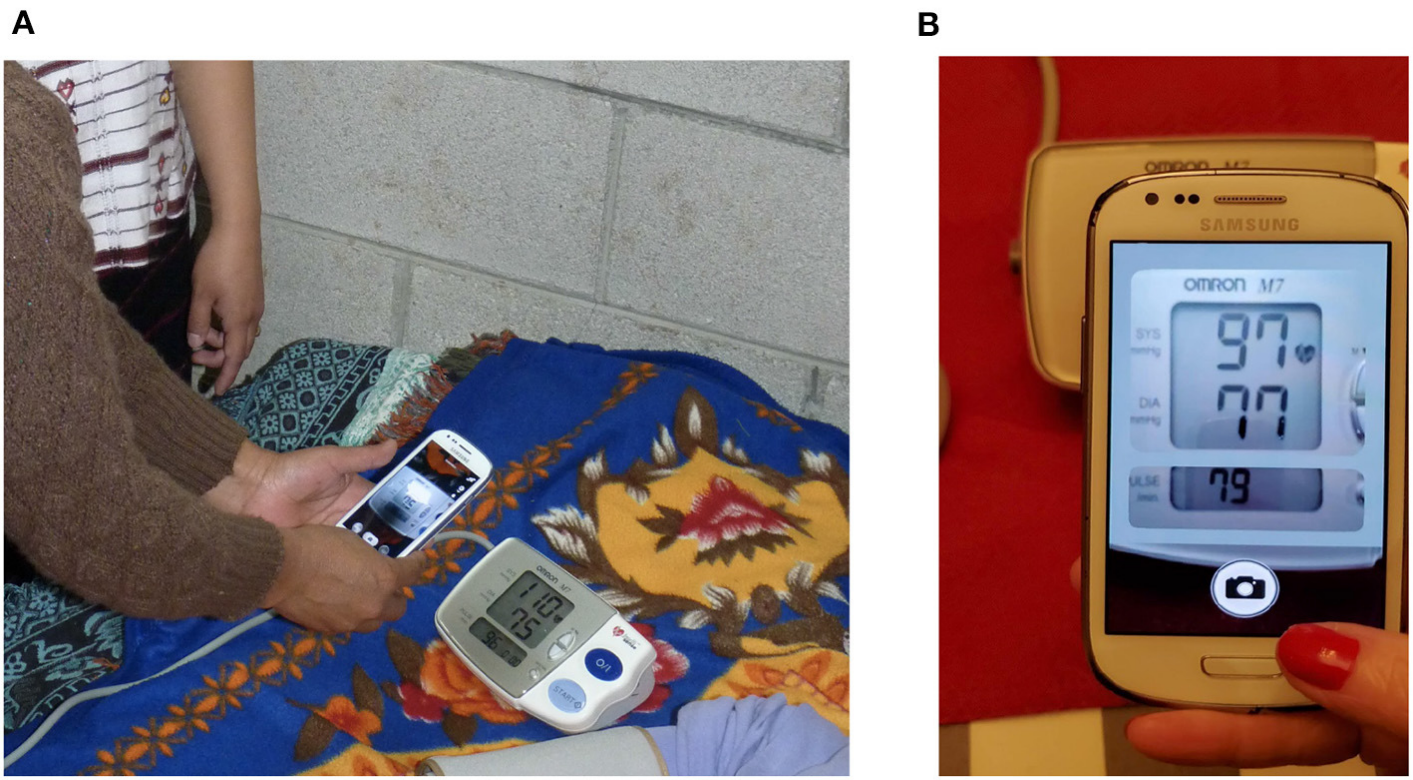
An Android-based app to capture blood pressure readings used in this study: **(A)** The app being used by traditional birth attendants in Highland Guatemala is shown (NBC Universal News Group, 2017). **(B)** The app interface as seen by the user is shown, with a “mask” to help align the liquid crystal display (LCD) and improve quality during capture.

**Figure 2:**
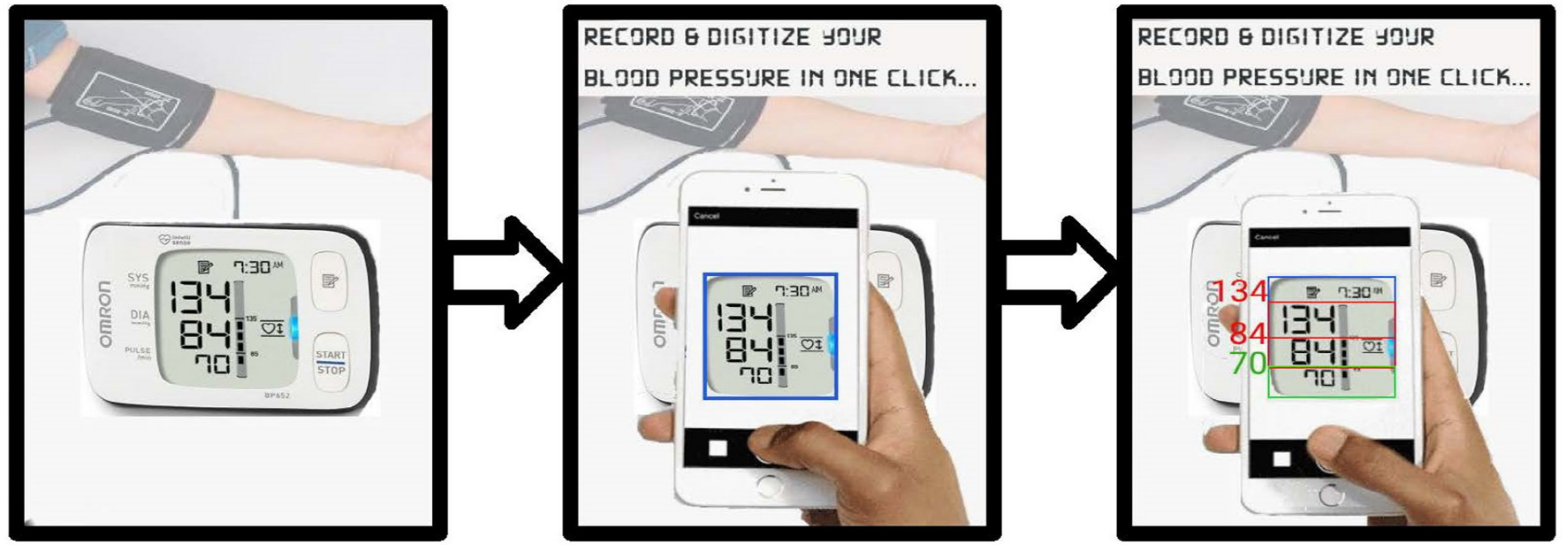
Steps of image transcription using cellphone camera.

In a recent step-wedge randomized control trial (RCT), the authors demonstrated that the introduction of blood pressure monitoring captured through an app led to improved outcomes in a mostly illiterate LMIC population (Martinez et al., 2018). Through this work, the authors intend to automate the existing manual transcription of blood pressure (It is important to note that the success of the proposed RCT was due to several factors to standardize blood pressure capture, which the authors address in more detail in the discussion). In the RCT, an Omron M7 (Omron Co., Kyoto, Japan) automated oscillometric BP monitor was used by traditional birth attendants in Highland Guatemala to screen pregnant women for hypertension and preeclampsia in rural settings. The data presented here was drawn from the RCT, and so represents highly realistic field data. The Omron M7 was chosen because it has been validated in a preeclamptic population (Bello et al., 2018). Figure 1A shows a traditional birth attendant capturing the data during a routine screening and a close-up of how the phone looks to the user during capture. BP readings were captured from the LCD screen using a standard cellphone camera and a bespoke Android app by traditional birth attendants during routine check-ups of patients (Figure 1B). Cellphone photographs of the display were used to train a deep learning approach to transcribe the readings into numerical values. An overview of the proposed approach can be seen in Figure 2.

## 2. Background on Number Digitization

Despite the increasing use of personal/electronic health records as well as smart and connected devices (e.g., via Bluetooth), the most widely employed method to record BP in clinical practice is through periodic manual transcription. Readings on automated BP devices are generally standardized with systolic and diastolic BP readings in large font and heart rate, date, time, and rhythm warnings in smaller letters (AHA, 2020). This provides spatial context to assist image capture into a useful digital format via OCR. Although transcription is performed on both paper and smartphone applications, both methods are prone to reporting erroneous readings due to transcription and legibility errors, and patient recall bias (Hall-Clifford et al., 2017). Hence, a number of BP data logging methods (with and without wireless data transmission) have been investigated to enable automated BP management of patients. Some of these involve memory card-based storage (Omron Healthcare Inc, 2012) and USB transfer to a computer using commercial data logger software (Microsoft, 2007; Omron Healthcare Inc, 2014), mobile-based data logging app using Bluetooth (Omron Healthcare Inc, 2020) or Wi-Fi connectivity (Withings, 2020). However, wireless and cable connections introduce complications that reduce the number of readings that can be captured. In earlier work, the authors showed that photos of medical data can help accurately capture such data (Hall-Clifford et al., 2017). This simple approach to logging BP readings using a smartphone app provides an easy, interactive, and convenient method using familiar technology.

There have been a number of OCR algorithms developed over the years, stretching back to the 1980s and 1990s, with a particular focus on machine learning approaches (Burr, 1988; Matan et al., 1992; Lecun et al., 1995; Kim and Govindaraju, 1997). Work has also focused particularly on number recognition (Leelasantiham, 2009; Babbar et al., 2018; Pham et al., 2018), building digital libraries through the process of extracting bibliographic data and inventorying details from book images (Kashimura et al., 1999; Chen et al., 2010), vehicular license plate recognition (Babbar et al., 2018), traffic sign recognition (Mammeri et al., 2014), and credit card number digitization (Leelasantiham, 2009). All these methods involve a pipeline of preprocessing, thresholding, delineation of area of interest using a template before finally applying character recognition in the localized region. Commercial OCR tools have generally been optimized for scanner-captured documents rather than camera-captured documents (Liang et al., 2004). For example, current PDF OCR tools include Google Drive OCR, Nuance, Adobe Acrobat Reader, and Readiris (Canon) (Pham et al., 2018). Image-based OCR tools include Tesseract OCR (Tesseract, 2005), Abbyy Mobile OCR Engine, and mobile applications such as CamScanner and My Edison (Mammeri et al., 2014). Although some of these applications offer rapid and low-cost digitization of data, their transcription accuracy decreases dramatically for images with geometrical distortions and noise due to image acquisition and environmental factors (Liang et al., 2004). Moreover, the lack of open research in these commercial systems makes assessment and repeatability of these approaches problematic.

Narrowing down the problem to only number digitization, there has been extensive research in handwritten digit recognition (Ali et al., 2019) as well as credit card, and street-view imagery (Leelasantiham, 2009; Goodfellow et al., 2014). Although a variety of classifiers have been used for this purpose, such as support vector machine, k-nearest neighbors and neural networks, convolutional neural networks appear to provide the best performance for digit recognition (Ali et al., 2019). In particular, Král and Čochner digitized analog gas meter readings using meter localization, perspective correction, and a digit-by-digit recognition using Linear Support Vector classification and template matching methods (Král and Čochner, 2015). However, very little research exists concerning the problem of LCD digit recognition. A relevant (non-peer-reviewed) computer vision project “Optimizer” developed by Izadi and Momeni (2018) used a deep learning approach to digitize gas pump readouts. In that work, the authors proposed a digit-by-digit as well as a multi-digit recognition approach (Goodfellow et al., 2014) to transcribe binarized segmented gas pump meter images using a convolutional neural network (CNN). Another project digitized gaspump meters on a digit-by-digit basis using a k-nearest neighbors approach (Kazmierczak, 2017). However, no statistics on how well these approaches perform were provided. Moreover, when these works were evaluated on the data in the study, they produced poor results.

Nevertheless, there is clear potential in modern CNN-based approaches, and in this work the authors propose an image-based OCR approach using CNN, which shares some similarities to the works presented by Goodfellow et al. (2014) and Izadi and Momeni (2018).

## 3. Methods

**Figure 3:**
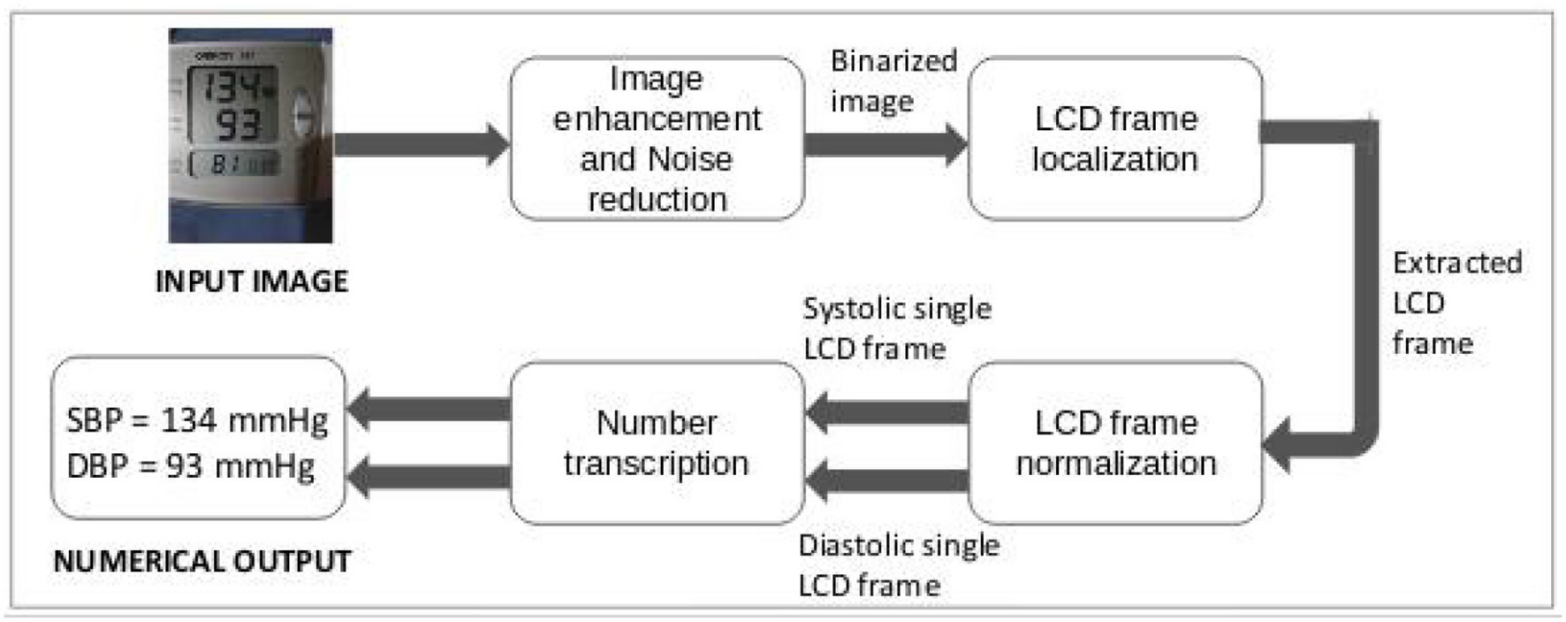
Design of proposed optical character recognition (OCR) approach to digitize blood pressure readings.

In this section, the step-by-step approach to convert BP monitor images into computer-readable numerical format is described, including localization, extraction, and recognition of the images. The end-to-end workflow can be found in Figure 3.

### 3.1. Database

Data used in this work were collected as a part of an RCT in collaboration with lay midwives on improving access to obstetrical care conducted in rural highland Guatemala in the vicinity of Tecpan, Chimaltenango. This trial was approved by the Institutional Review Boards of Emory University, the Wuqu Kawoq | Maya Health Alliance, and Agnes Scott College (Ref: Emory IRB00076231–“Mobile Health Intervention to Improve Perinatal Continuum of Care in Guatemala”) and registered on ClinicalTrials.gov (identifier NCT02348840). More details on the design and implementation of the data collection system, and the training of the traditional birth attendants can be found in Stroux et al. (2016), Martinez et al. (2017, 2018).

At each visit, a traditional birth attendant recorded at least two maternal BP readings using the Omron M7 self-inflating device (Omron Healthcare Europe BV, Hoofddorp, the Netherlands). With specific reference to the conditions for capturing images, all visits were conducted inside the mother's home, where lighting was generally poor, but highly variable. No prescription was given for adjusting light conditions or use of flash. The user was trained to align the image using a “mask” that appears to resemble the monitor (see Figure 1B), and retake if they were not happy with the result in terms of readability due to focus, lighting, cropping, or scale. The request to iterate until the users considered the images useful created an inflated representation of low-quality images in the given database compared to the number of visits, but also led to readable data for most visits. Each BP estimate was assessed on both of the subject's arms while the patient was in the supine position [The position was chosen to minimize changes in traditional practices as it produces a small offset in mean blood pressure, and reduces variability due to body habitus (Martinez et al., 2017)]. Once a BP reading was taken, the midwife registered the BP on a mobile app by taking a picture of the device screen. The mobile phone models used in this study were Samsung Galaxy S3 or J2. The matrix size/resolution of the images was 640 × 480 pixels. The spatial resolution depended on the distance of the camera from the blood pressure device. The size of the detected blood pressure LCD ranged from 137 × 146 to 264 × 303 pixels. The physical size of the Omron M7's number display is 2.5 × 2.5 cm (for the blood pressure) and 2.5 × 1.3 cm for the heart rate section. The numbers are 1.9 cm high by 1.25 cm wide for blood pressure and 0.64 cm high by 0.42 cm wide for heart rate.

Between January 2013 and July 2019, a total of 8,192 images were captured from 1,697 pregnant women carrying singletons between 6 weeks and 40 weeks gestational age. The systolic blood pressure (SBP), diastolic blood pressure (DBP), and heart rate (HR) of each BP image were manually transcribed by two independent annotators, storing the data in independent locations inaccessible to the other. Annotators screened each of the images for readability as well as image quality labels. Readability was defined as the ability to clearly transcribe the full numerical values of the SBP, DBP, and HR. If a value for any of these parameters could not be transcribed, one of the following labels was assigned to the image, which was then replaced as a “not a number” (NaN) during preprocessing.

- **Out of Focus, Fully Captured:** The image was out of focus/ blur, and it was not possible to identify SBP, DBP, as well as the HR values by a human. The image was annotated as “O.”
- **Contains Something Other than Blood Pressure:** The image contained something other than BP monitor but was not personally identifiable. The image was annotated as “N.”
- **Too Dark:** The image was too dark, and it was not possible to read SBP, DBP, and/or HR values by a human. The image was annotated as “D.”
- **Contains Reflections:** The image contained strong reflections due to illumination challenging the identification of its values. The image was annotated as “R.”
- **File is Corrupt:** The image file cannot be opened and was annotated as label “C.”
- **Contains Something Personally Identifiable Other than the Blood Pressure Data:** The image contained something other than the BP device screen that was personally identifiable—ear, eye, tattoo, identity card, fingerprint, etc. The image was annotated as “P.”

Sampling an average of four images per mother, distributed evenly over the 41 midwives who captured the data, a total of 7,205 images were annotated for the values of SBP, DBP, HR along with a quality label. The defined quality labels are as follows:

- **Blurred:** The image was out-of-focus/blur making it difficult to interpret values of SBP, DBP, and HR. The quality label given was “B.”
- **Dark:** The image lighting conditions were dark even if the values could be manually transcribed. The quality label given was “D.”
- **Contains Reflections:** The image contained reflections due to illumination variation, and reflection from LCD screen or from cellphone camera even if image is readable. The quality label given was “R.”
- **Far:** The BP monitor was excessively distant from the camera or zoomed out, leading to only a small region of the image/number of pixels representing the blood pressure reading. The quality label given was “FAR.”
- **Cropped:** The image LCD screen (region of interest) was cropped, but all the values were visible. The quality label given was “CROPPED.”
- **Good Quality:** The image had readable numbers without any quality issues described above. The quality label given was “OK.”

**Figure 4:**
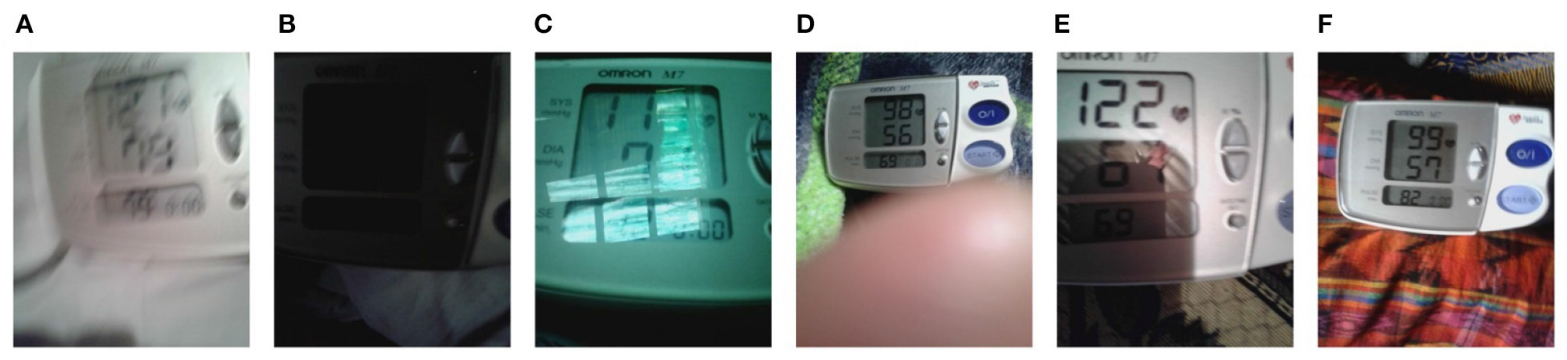
Examples of each class from blood pressure images dataset: **(A)** Blur. **(B)** Dark. **(C)** Contains reflections. **(D)** Far. **(E)** Cropped. **(F)** Good quality.

Examples of images with each quality label can be seen in Figure 4.

As the annotation process was manual, it may have been affected by typos and other human errors. To remove annotation errors, once the two annotators transcribed all the BP images, a third independent annotator reviewed those BP images in which the two annotators disagreed. Thus, the third annotator corrected any annotation error and generated the final spreadsheet used in this work. Segregation of these 7,205 images based on their quality metric yielded 740 “Blur” quality images, 314 “Dark” quality images, 3,885 images containing reflections, 375 “Far” images, 630 “Cropped” images, and 1,261 “Good Quality” images. Further, for the purpose of the analysis presented in this study, the authors categorized all these images into two categories: Good Quality images (Inclusive of images with “OK” quality label) and poor quality images (Inclusive of images with “Blur,” “Dark,” “Far,” “Contains Reflections,” and “Cropped” quality labels).

### 3.2. Preprocessing

Given the wide variability in the appearance of the BP monitor images due to orientation, zooming, environmental factors like lighting, shadows, noise, as well as image acquisition factors like motion, out-of-focus, and in-focus blurs as given in the previous section, preprocessing of the images was required before extracting the region of interest. The authors used OpenCV library (Bradski, 2000) for this purpose.

It can be noted that the digits on the BP LCD are not continuous (i.e., they are made of seven segments) and therefore have some similarities to halftone documents. Halftone documents are printed with one color of ink, in dots of differing size (pulse-width modulation) or spacing (frequency modulation), or both. This creates an optical illusion and when the half-tone dots are small, the human eye interprets the patterned areas as if they were smooth tones. As Adak et al. (2015) pointed out, classical binarization techniques on half-tone images do not produce the standard output for feeding into the OCR engine and need further processing. However, because the BP LCD more closely resembles text with artifacts of missing connectivity in a digit or letter, rather than half-tone documents, which have a more uniform missing pattern, we chose a different preprocessing approach described below.

#### 3.2.1. Image Enhancement

The first module of the proposed preprocessing algorithm involved enhancing the LCD frame boundaries in the image and thresholding it to enable accurate extraction of the BP and heart rate LCD frames from the images shown in the next module. For this, the image was first converted to grayscale. This step allowed faster processing of the images, saving on computation resources due to reduced size and number of channels. Next, it was fed to a bilateral filter to smooth the images while preserving edges (Bradski, 2000) followed by gamma correction to correct illumination levels in the image using non-linear transformation between the input pixel values and the mapped output pixel values given by (Bradski, 2000):

(1) $$\begin{array}{r} O={\left(\frac{I}{255}\right)}^{\gamma }*255 \end{array}$$

Binarization is the process of conversion of image using a threshold such that all of its pixels either take value 0 or 1. This step is essential in LCD frame extraction to obtain clearly defined frame boundaries as well as clearly defined digits contributing to overall accuracy of the OCR. Given the variance in image quality, the above adjustment regime was not enough for thresholding all images into their binary counterparts using a global threshold value. Hence, the authors decided to adopt the adaptive thresholding technique, wherein a threshold is calculated over small pixel neighborhood regions of image. Since different thresholds exist for different regions of the same image, this approach gave better accuracy for images with varying lighting and environmental conditions (Bradski, 2000).

#### 3.2.2. LCD Frame Localization

In this module, the BP and heart rate LCD frames were localized in the preprocessed image. Initially, the authors started inspecting simple contour attributes like width, height, center, size, and area. Due to orientation and zooming effects of the images, the size and the location of the LCD frames differed over a wide range in the preprocessed images. For example, the images annotated with quality label “FAR” had a small random portion of the image occupied by the BP monitor, while the images with quality label “CROPPED” had a cropped section of the BP monitor. In addition to that, the high amount of noise at the frame location due to environmental and image acquisition factors made the contour area unsuitable for LCD frame localization. Hence, in order to support the size attribute of the contours to localize LCD frames, the authors decided to inspect the aspect ratio of all the contours detected in the image. The aspect ratio of an object is the ratio of width/height of the object. Based on experiential analysis of these attributes for nearly 500 images chosen at random, the thresholds to localize the LCD frames were decided. Due to relatively smaller size of the heart rate LCD frame, the authors then corrected its 4 bounding box coordinates by verifying the corresponding 4 coordinates of BP LCD frame bounding box.

#### 3.2.3. LCD Frame Normalization

The obtained BP and heart rate LCD frames differed in sizes because of the differences in distance of the camera from the BP monitor. Hence, the authors normalized each of the frames to a fixed size using scaling. The bounding boxes extracted from the images included the boundary of the LCD. A simple approach to discard these boundaries by removing certain rows and columns along the boundary of the normalized LCD images was adopted. The row and column removal thresholds were decided through analysis on nearly 500 random images from the dataset, as validated by the work presented by Shah et al. (2009). Each of the BP LCD frames was further divided into half along vertical height to get systolic BP and diastolic BP LCD images. This sequence of single LCD binary images were fed to the number transcription model.

### 3.3. Number Transcription Using Convolutional Neural Networks

Transcription of medical device display values is a sequence recognition problem. Given the image of a medical device display, the task is to identify the BP readings in the LCD frame extracted from the image. These values are a sequence of digits where the accuracy of transcription depends on estimating the entire value and not individual digits independently. This is because a variation in a single digit has a significant effect on the estimated BP value proportional to the order of the magnitude of the digit. Hence, the authors based their system on the unified multi-digit recognition approach proposed by Goodfellow et al. (2014).

In the study, the authors proposed a CNN-based approach that simultaneously learned (*i*) the digits and (*ii*) where to look for them. The digits were then recognized based on the coverage at certain levels of accuracy obtained using a confidence threshold. The confidence threshold is the probability of the most likely prediction being correct. Thus, by representing the blood pressure value as a sequence of digits (*s* = *s*_1_, *s*_2_, ...*s*_*n*_), the aim was to train a probabilistic model of sequences given images. Hence, for output sequence *S* of *N* random variables (one per digit) given input image *X*, a probabilistic model *P*(*S*|*X*) would be learned by maximizing the log *P*(*S*|*X*) on the training data. Given that the maximum value of BP is a 3-digit number, the length of the sequence *s* was chosen to be 3. Also, since each of the digit variables could take a finite number of possible values (0–9), a softmax classifier could be used to get each of the digits, where input of classifier are the features extracted from *X* using the CNN. Using the back-propogation learning rule, the digit classifier would then generate the digits and not return anything if no digit is predicted. In this proposed study, a 180 × 80 input vector was fed to three-layer CNN with 32, 64, and 128 filters of dimension 5 × 5 to extract features from the corresponding feature vector. Each layer was followed by a batch normalization, ReLU activation, and maxpooling layer. The output feature vector from the CNN was then fed to a softmax classifier with three output channels, corresponding to the estimate for each of the three possible digits (Izadi and Momeni, 2018).

### 3.4. Experiments

The entire dataset was first balanced to create an equal number of systolic and diastolic single LCD frames. The high variance in the number of images of different quality meant that dataset balancing with respect to quality was not considered in current study. Both good-quality and bad-quality images were divided into training and test data in the ratio 3:1 to train and evaluate the performance of models developed in each experiment. A total of 542 good-quality images and 1,693 poor quality images comprised the test dataset, which were not used during any training or optimization.

#### 3.4.1. Experiment 1

In order to produce a baseline result to compare to the proposed approach, the authors used “Tesseract,” one of the most accurate open-source OCR engines. Originally developed at Hewlett-Packard in the mid 1980s, it has been maintained by Google since 2006 (Tesseract, 2005; Smith, 2007). Tesseract OCR is free and released under the Apache V2.0 open source license. No training was performed to optimize the parameters of the algorithm. However, the software was applied at each stage of preprocessing pipeline proposed in this work (as well as on raw data), and the best results were reported.

#### 3.4.2. Experiment 2

The authors also compared their proposed method with the commercial state-of-the-art model provided by Google for OCR to transcribe text from images known as Google Vision API (Google, 2020). While no public performance statistics are available for the Google Vision model, it is widely used by developers and therefore is perhaps the best “public” comparison with the proposed approach. It should be noted that Google vision API is not free for commercial use and it offers a limited number of API calls, after which payment must be made. This can be cost-prohibitive for many applications in low-resource contexts. Moreover, the algorithm requires processing in the cloud, which is not feasible in low resource regions of the world due to poor Internet connectivity issues, and may be illegal or unethical in a medical context. Again, the Google Vision API was applied to both raw and the preprocessed test images generated through the study, and the best performance was reported. No retraining of Google's API was possible. However, it was applied at each stage of the preprocessing and the best results were reported.

#### 3.4.3. Experiment 3

In this experiment, the proposed model was trained only on the good quality images by further dividing remaining good quality images into training and validation data in the ratio 3:1 and the best model was obtained by setting a model checkpoint on the incurred validation loss. The trained model was then tested on the held-out good quality images as well as the held-out poor-quality images to validate its performance on images of different quality.

#### 3.4.4. Experiment 4

In this experiment, the proposed model was trained on both the good-quality and poor-quality images by combining the remaining good-quality and bad-quality images together. The dataset formed was then divided into training and validation data in the ratio 3:1, keeping equal percentage of contributions from good- and poor-quality images. The best model was obtained by setting a model checkpoint on the incurred validation loss. The trained model was then tested on the held-out good-quality images as well as the held-out poor-quality images to evaluate its performance on images of different quality and compare its performance to the other approaches described in this work.

Finally, the CNN was also trained and tested with lighter architectures (one and two layers) to determine the effect of a more parsimonious architecture.

## 4. Results

### 4.1. Preprocessing

**Figure 5:**
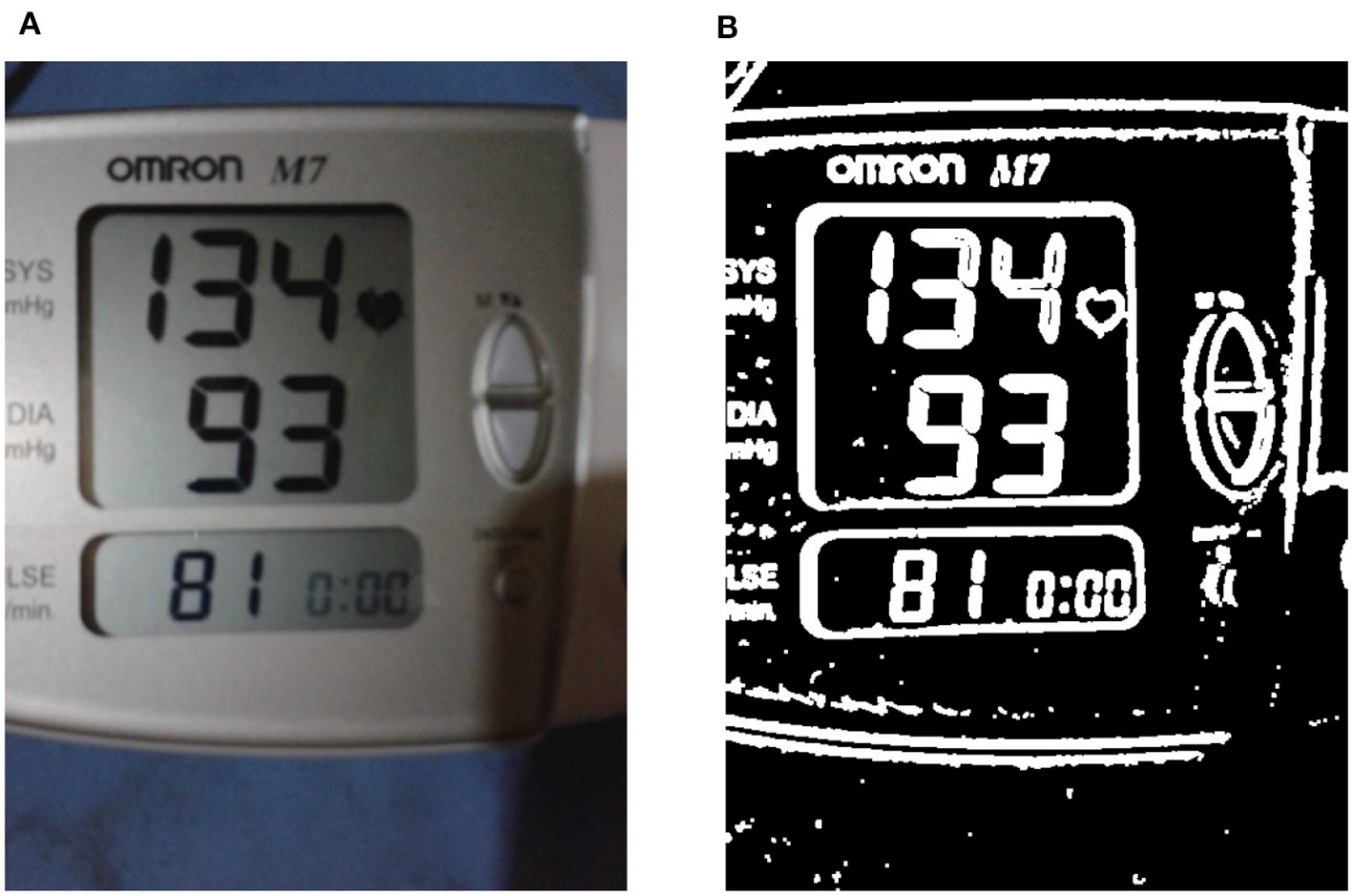
Enhancing the liquid crystal display (LCD) frame boundaries. **(A)** A sample input RGB image is shown, while in **(B)** the binary thresholded image obtained after performing image enhancement on the input is shown (see section 3.2.1).

**Figure 6:**
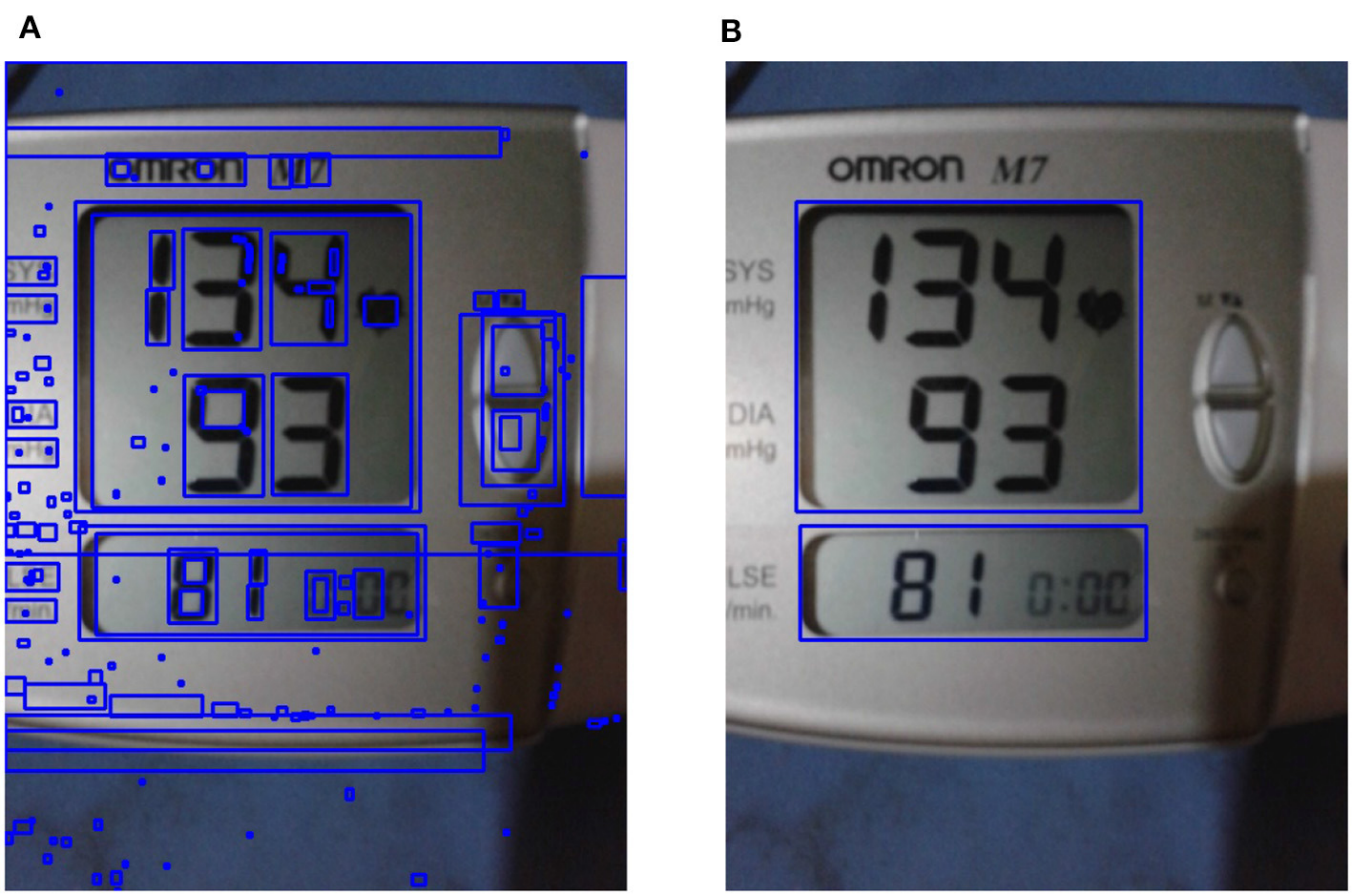
Liquid crystal display (LCD) frame localization. **(A)** All possible contours on enhanced image are shown; **(B)** the localized region of interest obtained using LCD localization are shown (see section 3.2.2).

**Figure 7:**
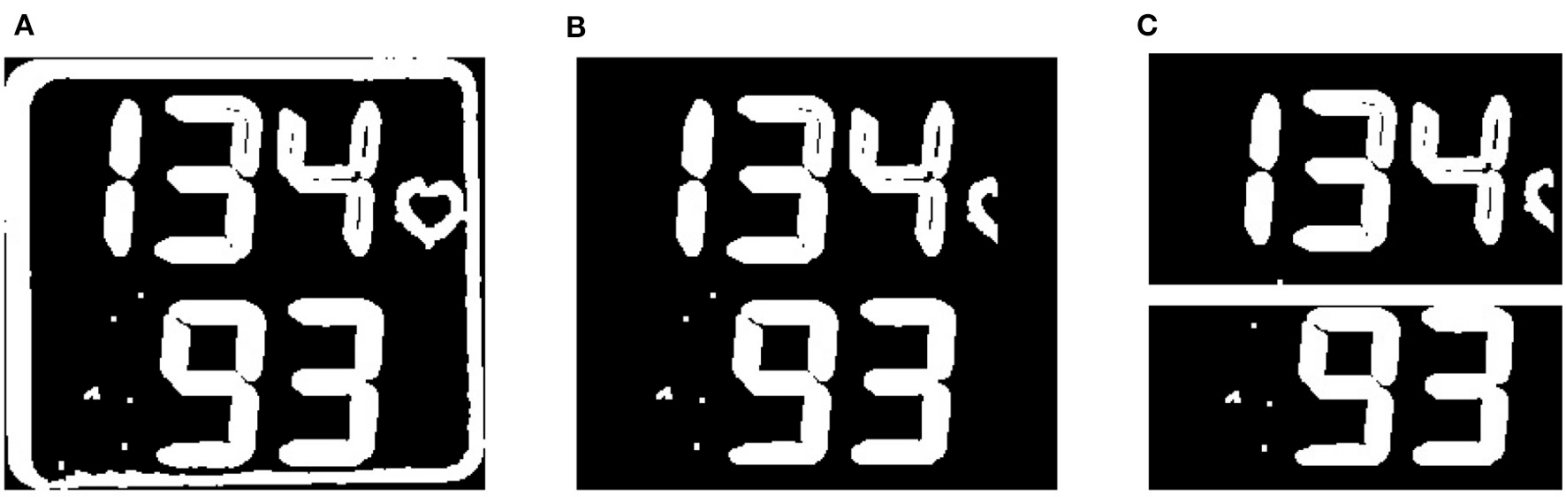
Liquid crystal display (LCD) frame normalization. **(A)** The binary thresholded blood pressure (BP) LCD frame extracted with contour border is shown, and **(B)** the LCD frame after border removal is shown. **(C)** 2 single monitor LCD frames as a final result of the LCD normalization module are shown (see section 3.2.3).

Given an input BP monitor image, the preprocessing module returned systolic and diastolic BP single monitor binary thresholded LCD frames. The process of obtaining the output through the serialized execution of 3 steps described in the previous section can be observed in Figures 5–7. An LCD frame extraction accuracy of 85% was observed after the preprocessing module on the good-quality images. On the other hand, only 57.8% of poor-quality images were extracted into their systolic and diastolic LCD counterparts.

### 4.2. Performance of Each Classification Approach

In experiments 1 and 2, Tesseract OCR engine and Google vision API were used to transcribe the held out test dataset, respectively. In experiment 3, the proposed model was trained on 1,082 good-quality single LCD images and was validated on 540 good-quality images to obtain the best possible CNN for the dataset. While in experiment 4, the model was trained on 5,020 images (all single LCD images except those in unknown test dataset) and its best possible solution was obtained through validation on 1,677 images (all single LCD images except those in unknown test dataset). For the lighter architectures, (one and two layers) we observed a 10–20% drop in accuracy and an increase in MAE of 3–8 mm Hg, indicating that the more complex architecture presented here is necessary, and that more complex architectures may improve performance.

Table 1 shows the results for classifying the images for the specified experiments. The results of experiment 1 demonstrates that Tesseract OCR engine accuracy is approximately 17% for good-quality and 7% for poor-quality images with pressure errors between 49 and 192 mm Hg. Experiment 2 shows that Google's Vision API can only achieve an accuracy of approximately 42 and 24% for good- and poor-quality images, respectively, with mean absolute errors of between 36 and 89 mm Hg. All of these estimates are far outside any acceptable bounds. This demonstrates that Tesseract and Google's OCR system are unable to produce usable results on the BP data and introduce significant errors into the digitization process. In contrast, the approach presented in this work (experiments 3 and 4) generally provide an acceptable performance. Experiments 3 and 4 resulted in similar accuracy rates for classifying good- and poor-quality images, although training with both good- and poor-quality images generally provided a marginal boost in performance on both types of images. Specifically, systolic and diastolic good-quality images obtained a higher accuracy around 90% for both experiments, whereas the poor-quality images yielded an accuracy around 63%.

**Table 1:** Optical character recognition (OCR) performance.

**Experiment**	**Training**	**Test data**	**Classification accuracy (%)**	**MAE (mm Hg)**
1	Tesseract	Held-out good-quality images (SBP)	20.2	185.2
		Held-out good-quality images (DBP)	14.3	49.3
		Held-out poor-quality images (SBP)	6.7	191.8
		Held-out poor-quality images (DBP)	7.9	52.8
2	Google Vision API	Held-out good-quality images (SBP)	42.1	64.03
		Held-out good-quality images (DBP)	43.2	36.46
		Held-out poor-quality images (SBP)	26.5	88.57
		Held-out poor-quality images (DBP)	23.0	56.47
3	CNN model, good-quality images	Held-out good-quality images (SBP)	88.1	**2.29**
		Held-out good-quality images (DBP)	86.1	1.73
		Held-out poor-quality images (SBP)	61.7	7.55
		Held-out poor-quality images (DBP)	62.8	5.03
4	CNN model, good- and poor-quality images	Held-out good-quality images (SBP)	**90.7**	3.19
		Held-out good quality images (DBP)	**91.1**	**0.94**
		Held-out poor-quality images (SBP)	65.1	8.00
		Held-out poor-quality images (DBP)	66.2	3.69

*For each experiment, the classification accuracy and the mean absolute error (MAE) is provided for the image sets. DBP indicates diastolic blood pressure and SBP indicates systolic blood pressure. Best performance statistics are in bold*.

## 5. Discussion

### 5.1. Comparison With Existing Methods

The method proposed in this work is a novel implementation for digitizing numbers on an LCD captured by independent devices such as a phone. Although the use of a CNN has previously been proposed for digitizing similar digits from gas meters, the work remains unpublished, except for a GitHub repository with sparse documentation and no assessment of performance. Moreover, the authors of this current work were unable to produce any useful results from the code provided via GitHub. As such, that work cannot be considered a predicate, and unfortunately, in the authors' experience, is representative of the state of much of the code posted publicly, in recent times.

**Figure 8:**
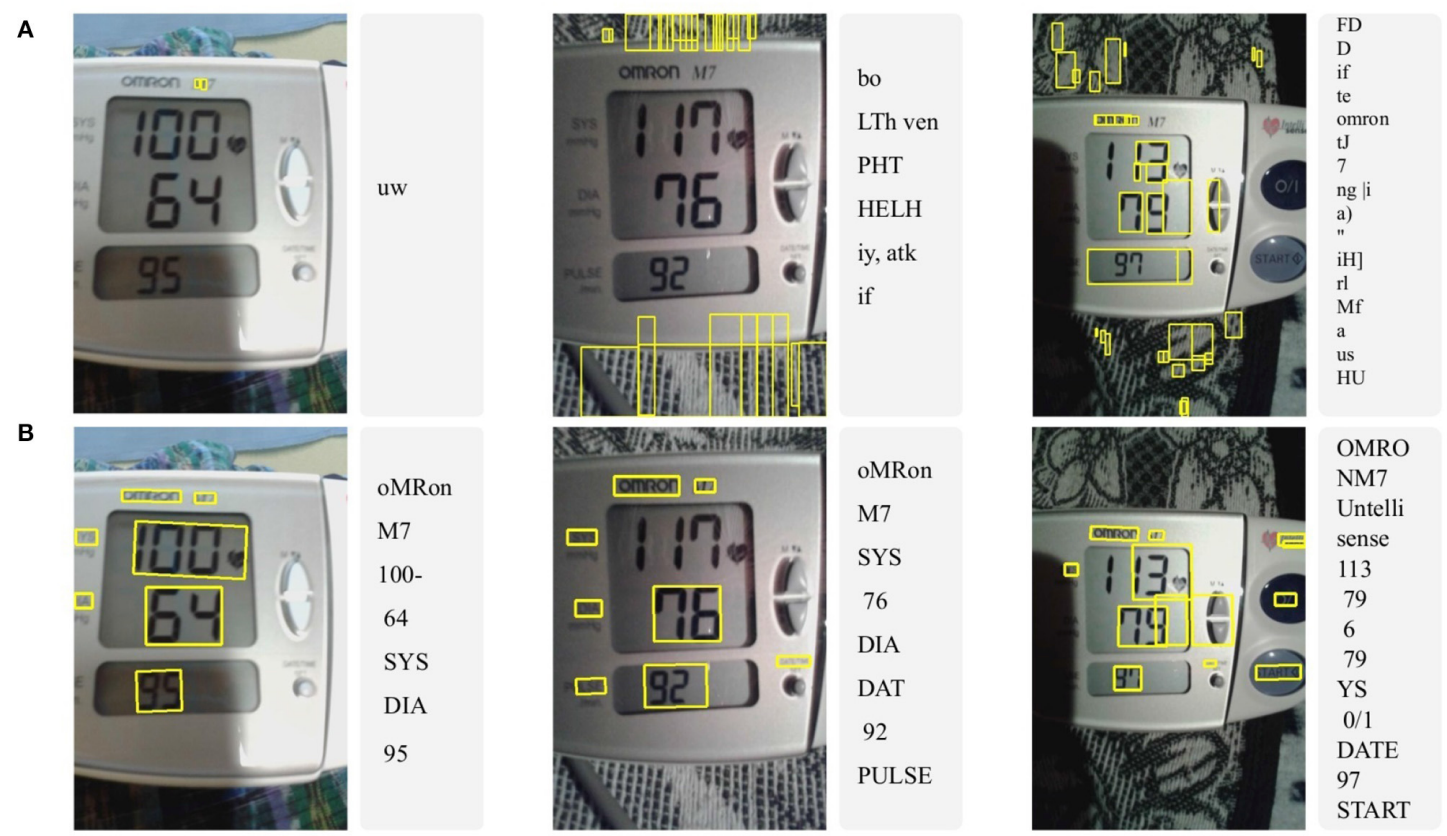
Three typical examples of optical character recognition (OCR) results using **(A)** Tesseract and **(B)** Google vision API on original (raw) good-quality images. Notice the significant errors produced by Tesseract with overwhelming false positive and negative detections, resulting in no useful information. The Google API produced acceptable results on only one of the photographs.

In this work, commercial state-of-the-art approaches were also tested. However, they produced unacceptable results with extremely large errors on the blood pressure images in this study. Figure 8 provides typical results, with transcription errors such as identification of non-digits as text (false positives), missing digits (false negatives), and inconsistent formatting of the text, making post-processing extremely difficult, or impossible.

### 5.2. Generalizability of Current Work

The method proposed in this article is highly generalizable and applies to a wide range of devices, beyond the blood pressure cuffs such as monitoring glucose level in diabetics populations (Low-cost blood glucose devices typically have LCD displays and no connectivity, which makes them well-suited for the application of the proposed method). The algorithmic complexity of the system proposed in this article is low enough to allow deployment on most modern Android smartphones using the TensorFlow Lite Android Support Library. The proposed method can therefore provide a first-line decision support mechanism for individuals or healthcare workers with little training. The connected nature of the phone can allow subsequent review to flag errors and provide a continually evolving and improving system.

A classic seven-segment digital number display is a standard format for many LCD interfaces on medical devices and different sizes of digits should not affect the analysis, since the CNN allows for scaling. However, there is a lower resolution limit where the phone may be too far from the device and the resolution would be too low. Also, BP numbers displayed in a color format would not affect the digitization process as the proposed method thresholds and converts the image into a binary image before feeding it to the model. However, for using blood pressure devices with fonts that differ greatly from the classic seven-segment number format, the network should be retrained.

### 5.3. Limitations of Current Work

The authors note several limitations of their work. Performance on low-quality data was poor, as expected—if the numbers in an image are cut off, or it contains substantial reflections that obscure the number, then there is little hope of an accurate transcription. The only way to correct such errors is at the point of capture. It is therefore important to develop an algorithm to identify the quality of an image that can run on the cellphone and alert the user to re-take the photo.

The authors also investigated the performance of the proposed method using different CNN structures. However, less complex networks degraded the results. More complex networks and more data may, therefore, improve the model performance and generalization to new images.

### 5.4. Future Work and Recommendations

Future work will be aimed at the development of image quality assessment using the extensive labeled database used in the study that can pre-select between unreadable and readable data, so that the system can feedback this information to the user. However, it is interesting to note that modern cameras such as the Google Pixel and the Samsung S10 series already have such software built-in. Therefore, in the coming years, as this technology trickles down to lower cost phones, there may be no need to develop additional methods, and the technology presented here could be integrated into future phones as standard, in a similar way to credit card number reading software is today.

As noted in the introduction, there are other issues that can affect BP accuracy in the field, including incorrect usage of the BP device, poor choice of device and arm cuff, poor body habitus, and transcription or transmission errors (particularly in low literacy populations). While the presented work only addresses the latter, the authors have demonstrated that the other issues can be mitigated with only a limited amount of training in a low literacy population (Martinez et al., 2018).

In particular, through a co-design process (Stroux et al., 2016; Martinez et al., 2017), the authors adapted the interface of the phone, and the training procedures to the local population's practices, such as patient assessment while supine. It is important to note that the success of the RCT was due to several factors in addition to standardized blood pressure capture, as a result of this preparatory fieldwork. This included building a multichannel communication modality (SMS, voice, GPRS, and Wi-Fi) linked to a coordinator who was able to deploy “care navigators” (Martinez et al., 2018). Nevertheless, the step-wedge nature of the assessment indicates that without the technology, manual transmission of information provided poorer outcomes.

## 6. Conclusions

This work provides a strong empirical analysis, which includes a significant amount of preprocessing to improve the quality of the images. The final method provides a low error for digitizing blood pressure, which is well within the FDA guidelines below 5 mm Hg (Ruzicka et al., 2016), making it suitable for general use.

In conclusion, the authors have presented evidence to show that the use of an app employing the methods described in this article may improve outcomes. However, an RCT may be required to more rigorously test this hypothesis. Since, a framework for such an RCT has been developed by the authors with the community with which the system was developed, it is hoped that the system can be implemented on a phone and its impact assessed in future work. To enable others to build off the work described in this article, the code and model has been made available under an open source license (Kulkarni et al., 2020).

## Data Availability Statement

The datasets generated for this study are available on request to the corresponding author.

## Ethics Statement

This work was part of a study approved by the Institutional Review Boards of Emory University, the Wuqu' Kawoq | Maya Health Alliance, and Agnes Scott College (Ref: Emory IRB00076231-Mobile Health Intervention to Improve Perinatal Continuum of Care in Guatemala) and registered as a clinical trial (ClinicalTrials.gov identifier NCT02348840).

## Author Contributions

SK and NK performed all the experiments and contributed to design of the system. GC designed the experiments, and managed the project. SK, CV, and NK curated and labeled the data, and contributed input to experimental procedures. PR and GC designed the data collection. All authors wrote and edited the manuscript.

## Conflict of Interest

The authors declare that the research was conducted in the absence of any commercial or financial relationships that could be construed as a potential conflict of interest.
